# Emerging Role of Quantitative Ultrasound-Based Imaging Techniques for Characterizing Rotator Cuff Tears: A Scoping Review

**DOI:** 10.3390/diagnostics13122011

**Published:** 2023-06-09

**Authors:** Andrew J. Nasr, Chris J. Pierson, Yi-Ting Tzen, Michael Khazzam, Nitin B. Jain, Yen-Sheng Lin

**Affiliations:** 1 Department of Applied Clinical Research, University of Texas Southwestern, Dallas, TX 75390, USA; 2 Department of Orthopaedic Surgery, University of Texas Southwestern, Dallas, TX 75390, USA; 3 Department of Physical Medicine and Rehabilitation, University of Texas Southwestern, Dallas, TX 75390, USA

**Keywords:** shoulder, rotator cuff, quantitative ultrasonography, ultrasound, shear wave elastography, sonography, musculoskeletal

## Abstract

Rotator cuff myosteatosis following cuff tears is very common and one of the most important prognostic factors in clinical management. Quantitative ultrasound-based imaging techniques (QUBIT) are frequently used along with magnetic resonance imaging (MRI) to evaluate rotator cuff fatty degeneration. However, the examination of rotator cuff tissue integrity by QUBIT is lacking a standardized imaging protocol and procedural methodologies. In this scoping review, we synthesized the current state of QUBIT against the reference imaging modalities in patients with rotator cuff tears. The literature search was extracted from 963 studies, with 22 studies included in the final review in accordance with the preferred reporting items for systematic reviews and meta-analyses extensions for scoping reviews. The selected studies included human participants and focused on measuring at least one prognostic or diagnostic factor using ultrasonography-based imaging with reference to MRI. The findings suggest both conventional B-mode ultrasound and shear wave elastography imaging were comparable to MRI-based imaging techniques for the evaluation of fatty infiltration and rotator cuff tear characterization. This review establishes guidelines for reporting shoulder-specific QUBIT aimed at developing a standardized imaging protocol. The objective was to enhance the diagnostic and prognostic capabilities of QUBIT in the clinical setting.

## 1. Introduction

Rotator cuff tears are highly prevalent, and an estimated 272,148 rotator cuff repairs were performed on an ambulatory basis in 2006 [1,2,3]. This amounted to more than $3 billion in direct surgical costs, which did not include expenses from diagnostic tests, office visits, and non-operative treatments such as physical therapy, medications, and injections [4,5]. Fatty infiltration (known as myosteatosis) of the rotator cuff muscles is an ectopic fat deposit that increases with aging and has been found to have a negative effect on tendon healing and on clinical outcomes [6]. The degree of myosteatosis is one of the most important prognostic factors in the operative and non-operative management of rotator cuff tears [7]. The incidence of irreparability of rotator cuff tears is 6.5–30% owing to tear size and fatty infiltration of the muscles [8].

In current clinical practice, risk stratification and surgical decision-making rely on outdated qualitative measurements of myosteatosis [9]. Using valid, reliable, and feasible quantitative imaging technology to evaluate myosteatosis has the potential to fill this void and can have a direct clinical impact at the point-of-care environment. However, the financial and economic impact of the use of diagnostic imaging procedures has substantially increased service demand in the healthcare system [10], due to longer life expectancy and patient expectations and preferences [11]. Magnetic resonance imaging (MRI) is the current standard for the assessment of fatty degeneration. MRI is also one of the most important contributors to healthcare costs in patients with rotator cuff tears [12]. Qualitative rotator cuff muscle atrophy and degenerative assessment using MRI often leads to inter-rater variability and potentially insensitive and inaccurate measurements of rotator cuff abnormalities [13]. Examination of the quantitative imaging at the point-of-care to assess rotator cuff muscle quality and hence its repairability is imperative to develop appropriate prognostic indications, inform care planning, and develop monitoring strategies [14].

**Figure 1:**
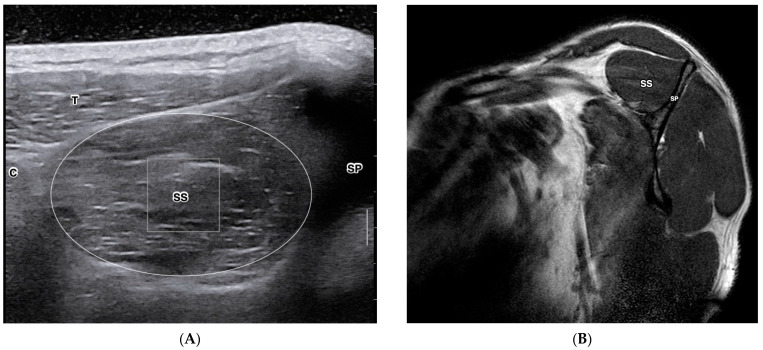
Cross-sectional view of the supraspinatus muscle of the right shoulder in a healthy 29-year-old female. Short-axis view of supraspinatus muscle in B-mode diagnostic ultrasound (**A**) and MRI T1 sagittal-oblique view (**B**). C—Clavicle; SP—Scapular Spine; T—Trapezius; SS—Supraspinatus. B. T1W MRI Y-view. SP—Scapular Spine, SS—Supraspinatus.

Quantitative ultrasound-based imaging techniques (QUBIT) are ultrasound technologies that derive multiparameter ultrasonographic metrics to quantify tissue properties. QUBIT is clinically accessible to quantify tissue changes induced by trauma, degeneration, healing, or tumors [15,16,17]. Studies on the QUBIT technique have shown promise in preoperative malignancy quantitative assessment for breast cancer [18], liver fibrosis [19], and thyroid lesions [20]. QUBIT has also been used to study musculoskeletal disorders, including rotator cuff tears and fatty degeneration [21,22]. Due to the nature of mechanical anisotropy and heterogeneity in musculoskeletal soft tissues, Cipriano et al. summarized the methodological challenges of establishing normative reference reporting of QUBIT in musculoskeletal soft tissues [23]. This study also suggested QUBIT-specific guidelines to standardize reporting procedures. However, there currently lacks a systematic approach to confirming rotator cuff tissue integrity using QUBIT techniques against the reference measures. Figure 1 depicts a cross-sectional view of the supraspinatus muscle on ultrasound (A) and MRI (B).

The overall purpose of this review is to synthesize imaging methodologies utilized for QUBIT and reference imaging modalities in patients with rotator cuff tears. The objectives are to (1) identify the current knowledge gaps of QUBIT in the literature, (2) compare QUBIT against reference measurements across all articles and within the rotator cuff tissue category, and (3) provide shoulder-specific guidelines for reporting QUBIT to improve the standardized protocol and enhance reproduction and replication, which are essential to advance the diagnostic and prognostic capabilities of this technology not only as a research tool but as point-of-care technology.

## 2. Materials and Methods

### 2.1. Search Strategy

A literature search was performed to review studies involving screening of rotator cuff fatty infiltration or tear characterization utilizing QUBIT compared with reference measures. The literature search was conducted according to the guidelines of Preferred Reporting Items for Systematic reviews and Meta-Analyses extension for Scoping Reviews (PRISMA-ScR) [24]. The literature search was conducted before December 2022 and utilized three databases: PubMed, Scopus, and OVID. The search was conducted using a combination of keywords related to ultrasound-based imaging techniques and rotator cuff fatty infiltration. For conventional ultrasound, the search keywords included those with “ultrasound” or “ultrasonography” and those with “sonography” or “ultrasound imaging”. For ultrasound elastography, the search keywords included “sonoelastography”, “elastography”, and “shear wave elastography”. For fatty infiltration, the search keywords included “fatty infiltration”, “fatty degeneration”, “myosteatosis”, “intramuscular fat”, “intramuscular adipose tissue”, “intermuscular fat”, and “intermuscular adipose tissue”. For rotator cuff, the search keywords included “shoulder”, “rotator cuff”, “supraspinatus”, “infraspinatus”, “teres minor”, “subscapularis”, and excluded “liver” or “cancer”. For searches on PubMed, the search keywords were replaced by the MeSH term.

The inclusion criteria included: (1) screening by ultrasound-based quantitative imaging; (2) diagnostic/screening tests to characterize rotator cuff tears with at least one reference measure; and (3) tests involved with and evaluated by human subject data. Studies were excluded if (1) the study population did not include evaluation of the rotator cuff in human subjects; (2) they were written in a language other than English; (3) they did not include at least one prognostic or diagnostic factor; or (4) they did not compare at least one ultrasound-based quantitative imaging modality to a current reference standard.

### 2.2. Data Extraction and Screening

The identified references were distributed to two authors (A.J.N. and C.P.), and abstracts were independently reviewed for inclusion. A standardized data extraction form was created by the research team. Two team members then used the pretested data extraction form to extract data from full-text articles. If there was disagreement about the inclusion of a study, a third reviewer (Y.-S.L.) was included for discussion. Finally, two authors (A.J.N. and C.P.) critically reviewed the full-text articles and summarized the findings. The following data were extracted for descriptive analysis and comparison in terms of imaging modality utilized, type of quantitative measurement, comparison to an established reference standard, and the identified prognostic or diagnostic factor explored.

### 2.3. Methodological Quality Analysis

Methodological quality was assessed for the studies that reported on quantitative metrics between ultrasound-based imaging modalities and the reference measure. The quality of the studies was independently assessed by two review authors (A.J.N. and C.P.). The included studies were systematically analysed using the Grading of Recommendations Assessment, Development, and Evaluation (GRADE) system of rating the quality of evidence [25]. The GRADE framework considers six domains that can influence the quality of the evidence and includes risk of bias, imprecision, inconsistency, indirectness, publication bias, and magnitude of the effect size. The risk of bias assessment was conducted in line with the NHLBI Risk of Bias Assessment Tool [26]. The starting point for the quality of the evidence was “high” for longitudinal studies that sought to confirm independent associations of the quantitative imaging variables between ultrasound and MRI. The evidence could decrease based on five factors: study limitations, inconsistency, indirectness, imprecision, and publication bias. Moreover, study findings with moderate or large effect sizes (i.e., lower limit of 95% confidence interval, odds ratio > 2.0 could lead to an upgrade in the quality of evidence). In total, all items across five categories for quality assessment were assessed: (1) study population, (2) assessment of imaging modality, (3) assessment outcome, (4) study design, and (5) quantitative data analysis. The criteria for each item were scored as “positive”, “negative”, or “not clear”.

## 3. Results

**Figure 2:**
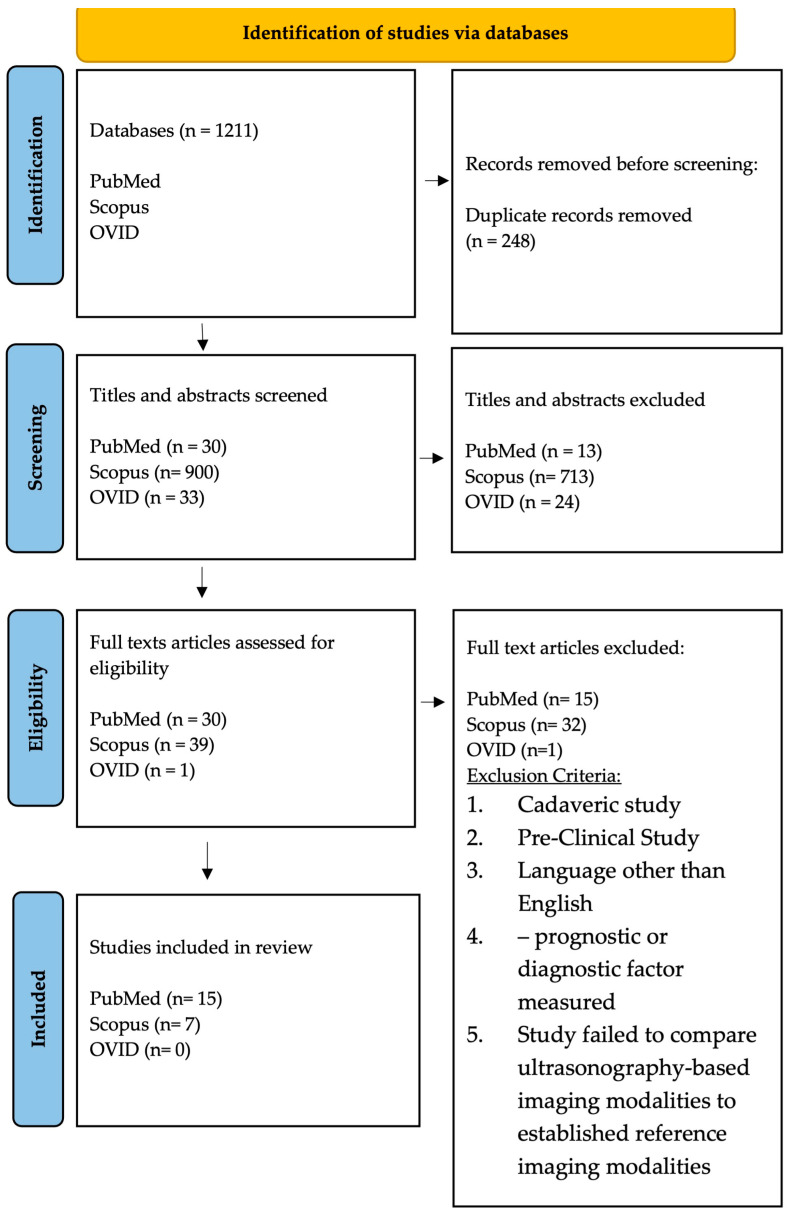
PRISMA-ScR flow diagram showing the article selection process.

A PRISMA-ScR flowchart diagram of the study selection process is shown in Figure 2. The search identified 963 primary studies without duplication from three databases. 750 articles were excluded after title and abstract reviews. Following 70 potential eligible articles being examined through full-text reviews, a total of 22 studies met inclusion criteria and were included in the final review. In total, six prospective studies, six retrospective studies, three cross-sectional studies, two case-control studies, and five case-series studies were included. Within the 22 articles, four ultrasound-based domains were studied: 8 studies characterized rotator cuff tears using shear wave elastography (SWE), 12 studies employed B-mode ultrasound (US), 1 study used 3D-US, and finally a single study used contrast enhanced ultrasound (CEUS). The characteristics of the rotator cuff tears and imaging modalities are presented in Table 1.

### 3.1. Participant Characteristics

A total of 1156 adult shoulders were examined with sample sizes ranging from 9 to 133 shoulders. Park et al. did not describe the gender breakdown of the 105 included subjects; otherwise, the collective studies included 564 male and 474 female subjects. Additional participant characteristics are detailed in Table 1.

### 3.2. Characteristics of Imaging Modalities

A summary of the imaging modalities and protocols can be found in Table 2. The transducers for rotator cuff imaging were linear, with frequencies ranging from 4 MHz to 18 MHz. MRI sequencing varied across all studies, ranging from proton density, T1 and/or T2-weighted sequencing. A more detailed description of ultrasound and MRI sequencing is presented in Table 2.

### 3.3. Assessment of Methodological Quality

A summary of the GRADE assessment of methodological quality is presented in Table 3. Overall, 20 of the 22 articles had low to no risk of bias based on the National Heart, Lung, and Blood Institute’s Risk of Bias Assessment Tool. Two articles had a moderate risk of bias due to a dropout rate greater than 30%. For overall quality of grading for the included studies, we adapted the criteria and only utilized five domains of the GRADE framework due to inconsistent and unavailable reports on effect size (Table 3). Three studies were graded 5, eleven studies were graded 4, followed by seven studies graded 3, and one study graded 2. There were disagreements about 20 out of 110 items, all of which were resolved by discussion to assess the total quality rating. The results were presented based on grouping the imaging modalities with diagnostic or prognostic factors.

### 3.4. Summary of Results

#### Fatty Infiltration Grading with Ultrasound and MRI

Park et al. performed a direct comparison of fatty infiltrate on ultrasound to MRI by collapsing the five-point Goutallier classification to a three-point dichotomous scale [27]. The agreement between MRI and US improved in both axes when using a dichotomous scale. However, the compressed imaging data may contribute to bias and inconsistency. Watanabe et al. quantified rotator cuff fatty infiltration and compared ultrasound and MRI to assess rotator cuff muscle using Goutallier’s MRI classification, ultrasound echo intensity of the supraspinatus muscle, and the echo intensity ratio of the supraspinatus muscle related to subcutaneous fat [28]. When compared to the Goutallier MRI classification stage, a significant difference was observed in echo intensities between the subcutaneous fat and the supraspinatus muscle. In addition, the echo intensity ratio of the supraspinatus muscle was significantly lower in Goutallier stage 0/1 than the other stages (stage 0/1 vs. stage 2, *p* = 0.004; vs. stage 3, *p* = 0.001; vs. stage 4, *p* = 0.001). This study suggested a clinically useful, objective, and quantitative assessment of fatty infiltration within the supraspinatus muscle. However, the quantitative assessment of other rotator cuff muscles such as the infraspinatus and teres minor were unclear, particularly in part due to the small sample size and retrospective study design. Seo et al. utilized the sonoelastography technique to quantify fatty degeneration and demonstrated high sensitivity, specificity, and accuracy in grading fatty degeneration [29]. Although sonoelastography showed good correlation with MRI and conventional ultrasound, the diagnostic criteria for fatty degeneration with the use of sonoelastography techniques are unknown. Wall et al. reported the diagnostic performance of ultrasound in grading fatty degeneration of the posterior and superior rotator cuff muscles by collapsing the Goutallier classification [30]. The agreement between the US and MRI scales in supraspinatus and infraspinatus muscles was excellent (0.71 < к < 0.83) but moderate in teres minor muscle (0.47 < к < 0.52). Despite the fact that the predictive values, sensitivity, and specificity showed satisfactory results, the authors acknowledged the rater’s reliability and introduced bias due to the reduction of the rating scale being unknown. Khoury et al. compared sonography with MRI for the evaluation of fatty infiltration within the supraspinatus muscle. This study also investigated the ability of US to quantify muscle atrophy with the evaluation of the occupation ratio of the supraspinatus muscle [31]. Although conventional sonography compared with MRI showed good correlation to assess both supraspinatus muscle atrophy and fatty infiltration, the reproducibility and generalizability of these measures to other rotator cuff muscles were unclear. This review included four studies that investigated the accuracy of ultrasound imaging to detect the rotator cuff tear and compared it with MRI as a reference measure. In these studies, no statistical differences were found in diagnostic accuracy between these two modalities [33,34,35]. While these studies compared the diagnostic accuracies of rotator cuff tears between ultrasound and MRI, the prognostic capacities of quantitative ultrasound-based imaging modalities in the asymptomatic or early stages of the symptomatic phase remain to be investigated.

For the quantitative assessment of the rotator cuff muscle thickness, Ueda et al. demonstrated the agreement between the cross-sectional muscle thickness of the rotator cuff muscles on MRI and ultrasonography [36]. Significant correlations were found for the three rotator cuff muscles: supraspinatus, r = 0.67, *p* < 0.001; infraspinatus, r = 0.63, *p* < 0.001; and teres minor, r = 0.61, *p* < 0.001. However, the cross-sectional area has been shown to be prone to underestimating the muscle atrophy at whole muscle levels [49]. Kretic et al. performed a reliability analysis of muscle thickness in patients with ruptured and intact supraspinatus tendons [37]. For the rupture group, the occupation ratio measured by US and MRI had a Pearson correlation coefficient of r = 0.640, and for the non-rupture group, r = 0.611. When the two groups were combined, r = 0.912. Although US measurements are not as strongly correlated as the cross-sectional area and occupational ratio measured by MRI to quantify muscle atrophy, US is still considered the first imaging modality to describe supraspinatus muscle atrophy and fatty degeneration. Yi et al. investigated the relationship between supraspinatus thickness measured by ultrasound and cross-sectional area measured by MRI in both healthy and hemiplegic patients. The Pearson correlation coefficient when muscle thickness on ultrasound was measured at the scaplar notch and at the Y-view on MRI was r = 0.72. When muscle thickness was measured at the scapular notch in both US and MRI, r = 0.76 [38]. Chen et al. found medium to large effect sizes (r = 0.4–0.8) for correlating the large-to-massive rotator cuff tears and good predictive validity for surgical reparability between B-mode ultrasound and MRI findings [39]. Despite the promising findings on reliability in the examination of these measurements, the small sample size of patient populations and the lack of high quality and comprehensive quantifications of rotator cuff muscle tissue properties were not included.

Eight studies utilized SWE to compare quantitative characteristics of the rotator cuff and correlated the findings with MRI, with conflicting results. Huang et al. found that SWE-derived rotator cuff muscle stiffness is highly correlated with rotator cuff tear size and severity [40]. In addition, shear modulus values of supraspinatus and infraspinatus muscles among affected shoulders were significantly stiffer than the values in the contralateral normal shoulder. However, the shear modulus values within each region of interest were qualitatively determined without quantitative measures. Jeong et al. utilized SWE as a prognostic marker in patients with rotator cuff tears and found that patients with insufficient repair exhibited higher mean Goutallier grade, mean muscle atrophy grade, mean supraspinatus elasticity, mean elasticity ratio, and mean gray-scale fatty infiltration grade and showed a lower mean occupation ratio [42]. While the strong inter- and intra-observer agreement between SWE and MRI measures demonstrated promising prognostic markers for preoperative evaluation of fatty infiltration, the generalizability of the complementary prognostic markers of preoperative evaluation to other patient populations remains to be investigated. Krepkin et al. found a statistically significant negative correlation between shear wave velocities in anteroposterior measurement of tear size in the middle (r = −0.79) and the mean region of interest (r = −0.68) [43]. A significant negative relationship was also found among shear wave velocities, the sum of anterroposterior, and the degree of retraction measurements in the middle region of interest (r = −0.72). However, the matching criteria of regions of interest, sensitivity, and specificity between ultrasound and MRI were not described, nor was it described whether quantitative elastography is able to detect subtle changes of tendon degeneration. Itoigawa et al. found the strongest correlation of stiffness with the shear wave modulus to be the posterior deep muscle of the supraspinatus (r = 0.69) [46]. The correlation of stiffness with the Goutallier stage on MRI was weak (r = 0.48). Although there is a high correlation between ultrasound SWE and MRI measures, the length of the retracted supraspinatus tendon may influence the elasticity measured by SWE.

In terms of controversial findings to interpret the SWE findings with caution, Ruder, Lawrence, and their colleagues reported findings as the lack of statistically significant predictability of the SWE-estimated shear modulus with pre-surgical MRI variables of tear characteristics and repair integrity [41,45]. Rosskopf et al. found decreased shear wave velocities were associated with increased fat content, degrees of muscle atrophy, and tendon retraction, but not tendon integrity [44]. Gilbert et al. found a strong positive correlation (r = 0.82) between SWV measured with SWE and the fat-to-water ratio on MRI analysis [47]. However, the reproducibility of the study findings measured by SWE and spectroscopic measuring techniques was susceptible to observer-dependent bias, inhomogeneous ROIs, and a lack of biological references such as histological quantification. Kunz et al. reported a strong correlation (r = 0.67) between preoperative profusion rate on contrast-enhanced ultrasound and MRI with a 6-month follow-up reassessment of the supraspinatus tendon [48]. Although patients with repaired tendons showed significant preoperative supraspinatus perfusion within the 4th quartile at follow-up, the patients with perfusion within the 1st quartile presented with tendon retears. To establish rigorous quantitative ultrasound-based imaging as a meaningful and valid diagnostic and prognostic tool, standardized protocols, and methodologies need to be employed in future studies. Our group provides recommendations for standardization and reporting of relative methodological practices in Table 4.

## 4. Discussion

A prerequisite to quantitative ultrasound disrupting current clinical decision-making patterns is a standardized protocol and methodological reporting. The primary aim of this scoping review was to provide a comprehensive list of methodological reporting recommendations. By employing standardized methodologies in quantitative ultrasound-based imaging, this study serves as the basis for future work involving an umbrella review and meta-analyses for a more comprehensive evaluation of the utility of quantitative imaging tools.

Quantitative ultrasound-based imaging provides objective measures for assessing the characteristics of rotator cuff pathology. Emerging imaging techniques, such as shear wave elastography imaging, are an advanced ultrasound-based modality that has seen increased utilization for better understanding musculoskeletal disorders. Despite the growing interest, guidelines for standardized protocols and clinical assessment of rotator cuff structures to incorporate current clinical practice are lacking. Currently, there is no consensus on the standardization of research methodology for the use of SWE imaging to investigate rotator cuff pathology. Prior studies have shown heterogeneity in the clinical utilization of quantitative ultrasound imaging. However, there is a lack of comparative studies to synthesize characteristic findings between quantitative ultrasound-based imaging and reference measures. To the best of our knowledge, this scoping review is the first to systematically synthesize the use of quantitative ultrasound-based imaging applications to characterize rotator cuff integrity. Future studies based on this interdisciplinary review and discussion will facilitate the development of a standardized ultrasound-based study protocol related to quantitative imaging.

Both conventional B-mode ultrasound and shear wave elastography had strong agreement compared to MRI-based imaging techniques for evaluating the grading of fatty infiltration within rotator cuff muscles. The level of agreement was strongest for the supraspinatus and infraspinatus, and the level of agreement diminished for the teres minor [30]. In addition, strong associations were found when utilizing occupation ratio as the metric for grading muscle atrophy, with a correlation coefficient of 0.90 [31]. Quantitative ultrasound also shows good utility in full-thickness rotator cuff tear detection. Previous studies investigated the accuracy of ultrasound imaging and found no statistically significant difference in diagnostic accuracy compared to MRI [33,34,35]. Studies also found strong associations between B-mode ultrasound and MRI in the measurement of muscle thickness, with correlation values ranging between 0.61 and 0.912 [36,37,38]. SWE is the emerging ultrasound-based quantitative imaging technique and has continued to show promise in the last decade. In six of the eight studies, strong correlations were found between repairability of rotator cuff tears and tear size associated with SWE-derived measures of tissue stiffness. However, Ruder and Lawrence did not find significant associations between SWE-derived measures of stiffness and predictability of post-operative function, tear size, tendon retraction, occupation ratio, or fatty infiltration, respectively. One of the potential explanations for the contradictory findings is attributed to the fundamental difference in medical imaging and technical constructs between SWE and MRI [41,45]. Shear-wave elastography can assess various aspects of tissue properties, whereas MRI-based imaging assesses structural and compositional changes or chemical shifts within the tissues [50,51,52]. Based on findings from an in vitro study, there appears to be clinical promise in combining MRI and SWE in the treatment planning for patients with rotator cuff tears [21]. Contrast-enhanced ultrasound imaging was underrepresented in this scoping review and shows great promise in musculoskeletal applications. Kunz and colleagues found a strong correlation between profusion rate and the healing status of the supraspinatus. Patients with higher profusion rates had a greater likelihood of an intact repair at 6 months. However, the relevance of the findings was hampered by a small sample size and insufficient power, partially due to the higher attrition rate of enrolled patients who completed the follow-up assessments. Future studies using contrast-enhanced ultrasound imaging are needed to increase the clinical utilization and quantitative promises of these imaging measures.

Quantitative ultrasound-based imaging is inherent to conventional B-mode ultrasound measures: rater dependence and steep learning curves. Investigators need to be keenly aware of patient position throughout ultrasound imaging, as any volitional movement or tension on the muscle of interest can skew findings. 21 of the 22 authors reported patient positioning during ultrasound examination; providing a detailed description of patient position is paramount to not only the reproducibility of the methods but also to minimize bias during data collection. Nevertheless, there are advantages to this type of imaging modality, including the absence of ionizing radiation, cost-effectiveness, and real-time application with subsequent interpretation of findings during functional examinations. Standard B-mode, SWE, and CEUS had similar findings when compared to a reference measure. B-mode ultrasound had similar diagnostic accuracy to MRI as well as the ability to measure fatty infiltration and muscle cross-sectional area. SWE, similarly, was comparable for quantifying fatty infiltration, but results were mixed for quantifying stiffness of the rotator cuff muscles and tendons. The CEUS profusion rate had a strong correlation with the integrity of the repaired supraspinatus tendon with MRI findings. Overall, quantitative ultrasound evaluation of rotator cuff tears shows promise, but standardized reporting of methodology will allow for more rigorous assessments of its scientific merit and clinical utilization. As evident from the GRADE Framework, 8 of the 22 studies had overt limitations in study design, data collection, data reporting, and/or other factors leading to a grade of less than or equal to 3 (out of a possible 5).

Rotator cuff tears remain a highly prevalent musculoskeletal condition and account for more than $3 billion in direct surgical costs [1,2,3]. Costs associated with diagnostic imaging are excluded from these cost estimates [4,5]. A 2017 study found the mean cost to the consumer of a shoulder MRI was $1874 (ranging from $500 to $4000) [53]. Risk factors associated with rotator cuff disease remain mostly unmodifiable, including but not limited to age, genetics, and trauma [54]. A previous review concluded that the comprehensive analyses for evaluating fatty infiltration of the rotator cuff involved mostly qualitative or quasi-quantitative methodologies [55]. Given that the presence of fatty infiltration is a strong predictor of poor outcome in patients undergoing rotator cuff repairs, cost-effective diagnostic tools for quantitative evaluation of fatty infiltration at the bedside can greatly improve the management of this patient population. The emergence of quantitative ultrasound-based imaging as a point-of-care tool can improve the decision-making process in the management of patients with rotator cuff disease. Quantitative imaging remains a vital tool in designing more rigorous musculoskeletal research protocols compared to qualitative or quasi-quantitative methods. Establishing ultrasound-based imaging as a reliable and valid tool will reduce the financial burden on the healthcare system in the U.S. To address the limitations within the current literature, this study provides thorough reporting recommendations for the use of QUBIT in the examination of shoulder-related conditions. The focus of these recommendations is to standardize the reporting of participant demographics, study methodologies, imaging protocols, and imaging results with the goal of maximizing the reproducibility and clinical significance of future studies.

This study has several limitations associated with the current state of quantitative ultrasound imaging research. most notably the inconsistent reporting of study methodology. As evident in Table 3, many studies did not provide a detailed imaging protocol for ultrasound or reference imaging. Secondly, the current level of evidence is primarily case-series, case-control, or retrospective studies, each with inherent limitations. Final validation of these new imaging modalities requires more rigorous investigation with large sample sizes and objective metrics. Thirdly, most published data involved studies lacking a sample size justification, and no study has employed the highest level of study design, such as a randomized clinical trial in a longitudinal setting. Finally, given the technical nature and operator dependence of ultrasound imaging, reliability assessments are warranted to inform comparative effectiveness. To date, quantitative ultrasound-based imaging for the assessment of rotator cuff tears remains an early-stage exploratory modality, and the need to validate these tools in the clinical setting remains a glaring gap in the literature.

## 5. Conclusions

This scoping review synthesizes the current state of the literature on the use of QUBIT for assessing rotator cuff pathology. The review of the literature found that both conventional B-mode ultrasound and SWE imaging were comparable to MRI-based imaging techniques for the evaluation of fatty infiltration and rotator cuff tear characterization. SWE is an emerging form of QUBIT and shows great promise in the quantitative evaluation of rotator cuff characteristics and fatty infiltration. Future work, including emerging radiomic features extracted from multi-parameter MRI, ultrasound-based SWE, and an artificial intelligence approach, is warranted to predict clinically significant rotator cuff lesions and distinguish the rotator cuff pathology phenotypes.

## Figures and Tables

**Table 1:** Summary of included articles.

Study	Study Design, Evidence Level	N (Sex M/F)	Patient Demographics	Imaging Modality	Purpose	Results
B-Mode Ultrasound and MRI						
Park [27]	Diagostic, I	108 (Unknown)	Patients with shoulder pain with suspected rotator cuff pathology	US and MRI	Correlating US echo intensity to Goutallier’s Classification	US grading of the infraspinatus based on short-axis architecture. Good agreement for both supraspinatus (k = 0.63) and infraspinatus (k = 0.68) in long- and short-axis scans, echogenicity showed moderate agreement for supraspinatus (k = 0.51) and infraspinatus (k = 0.50).
Watanabe [28]	Restrospective Cohort, III	27 (15/12)	Patients with shoulder pain with suspected rotator cuff pathology	US and MRI	Correlating US echo intensity to Goutallier’s Classification	Tukey’s Multiple Comparison showed significant differences in echo intensity on Stage 4 compared to other Goutallier stages (*p* < 0.05) implying US may be able to quantitatively assess fatty infiltration of the supraspinatus muscle.
Seo [29]	Retrospective Cohort, III	101 (56/42)	Patients with shoulder pain with suspected rotator cuff pathology	US and MRI	Correlating US echo intensity to Goutallier’s Classification	Sonoelastography is valuable in the quantitative assessment of the severity of the fatty atrophy of the supraspinatus and has an excellent accuracy (91%), and almost perfect agreement (weighted kappa k = 0.81) with MRI findings.
Wall [30]	Prospective Cohort, II	80 (46/34)	Patients with shoulder pain with suspected rotator cuff pathology	US and MRI	Correlating US echo intensity to Goutallier’s Classification	Agreement between MRI and ultrasonography was substantial for the supraspinatus and infraspinatus (k = 0.78 and k = 0.71, respectively) and moderate for the teres minor (k = 0.47)
Khoury [31]	Case-Series, IV	45 (19/20)	Patients with shoulder pain with suspected rotator cuff pathology	US and MRI	Correlating US echo intensity to Goutallier’s Classification	Good correlation (r = 0.90) between sonography and MRI for the assessment of supraspinatus muscle atrophy and fatty infiltration.
Chen, X [32]	Prospective Cohort, II	52 (28/24)	Patients with k-wn full-thickness rotatro cuff tears with pending surgery	3D US and MRI	Diagostic Accuracy of Rotator Cuff Tear	Overall diagnostic accuracy of 3D-US in evaluating RCT patterns (82.7%) was significantly higher (*p* = 0.008) than that of the MRI (57.7%).
Guerini [33]	Retrospective Cohort, III	15 (7/8)	Patients with suspected lesion(s) of rotator cuff tendons with posterior pain in the infraspinatus fossa	US and MRI	Diagostic Accuracy of Rotator Cuff Tear	Tears at the myotendinous junction of the infraspinatus can be diagnosed on US examination (100% accuracy compared to MRI).
Chen, D [34]	Retrospective Cohort, III	36 (3/33)	Patients with Rheumatoid Arthritis	US and MRI	Diagostic Accuracy of Rotator Cuff Tear	US and MRI yielded comparably high accuracy for full-thickness tears 92.9% and 96.4%, respectively but US detected only 62.5% of partial tears compared to 87.5% on MRI.
Rutten [35]	Retrospective Cohort, III	68 (37/31)	Patients with Shoulder Pain	US and MRI	Diagostic Accuracy of Rotator Cuff Tear	There was comparably high accuracy for detecting full-thickness RCT with ultrasound (78%) and MRI (79%) as compared to surgical findings.
Ueda [36]	Case-Series, IV	47 (26/21)	Patients with Rotator Cuff Tears	US and MRI	Muscle thickness on US and correlating to CSA on MRI	Significant correlations between the measurements of muscle thickness of the rotator cuff by MRI and US (SSP: r = 0.67, ISP: r = 0.63; TM: r = 0.61).
Kretic [37]	Prospective Cohort, II	87 (33/54)	Group 1-43 patients with complete tendon rupture/full-thickness tear Group 2-control group 44 patients without tendon rupture	US and MRI	Muscle thickness on US and correlating to CSA on MRI	Ultrasound supraspinatus muscle thickness measurement is highly correlated with MRI measured occupation ratio (r = 0.853) and MRI measured cross-sectional area (r = 0.890).
Yi [38]	Case-Series, IV	20 (13/7)	10 -rmal Subjects and 10 Patients with Hemiplegia	US and MRI	Muscle thickness on US and correlating to CSA on MRI	Supraspinatus thickness measurement by US is positively correlated (r = 0.72) with the CSA of the supraspinatus muscle on MRI.
Chen, P [39]	Cross-Sectional, III	50 (29/21)	Patients with large-to-massive rotator cuff tears	US and MRI	US to examine tear status and correlated to MRI	Correlation coefficients medium to large effect sizes (r = 0.4–0.8). Heckmatt scale for infraspinatus muscles was the most accurate ultrasound predictor.-significant differences in AUCs among the MRI and ultrasound predictors were found.
Shear wave Elastography (SWE) and MRI						
Huang [40]	Prospective Cohort, II	133 (80/53)	97 Patients with rotaotr cuff tears and 36 control patients	SWE and MRI	SWE to examine tear status and correlated to MRI	Severely fatty-infiltrated rotator cuff muscles (Goutallier stage 3–4) possessed a significantly higher Δshear modulus compared less fatty-infiltrated muscles (Goutallier stage 0–2) (*p* < 0.001). Muscles with more distinct tendon retraction (Patte 3 vs. Patte 1–2) and more obvious muscle atrophy (Tangent sign+ vs. −) were also significant (*p* < 0.001).
Ruder [41]	Prospective Cohort, II	23 (16/7)	Patients undergoing arthroscopic rotator cuff repair	SWE and MRI	SWE to examine tear status and correlated to MRI	Presurgical shear modulus generally did not improve the prediction of functional outcomes above and beyond that provided by MRI variables alone (*p* > 0.22)
Jeong [42]	Prospective Cohort, II	74 (37/37)	Patients undergoing rotator cuff repair	SWE and MRI	SWE to examine tear status and correlated to MRI. Predictability of successful repair	Patients with insufficient, versus sufficient, repair exhibited higher mean Goutallier grade, mean muscle atrophy grade, mean supraspinatus elasticity, mean elasticity ratio, and mean gray-scale fatty infiltration grade and showed lower mean occupation ratio. AUC for predicting insufficient repair was 0.945 for Goutallier grade, 0.961 for occupation ratio, 0.900 for muscle atrophy grade, 0.874 for mean elasticity, 0.971 for elasticity ratio, and 0.912 for gray-scale fatty infiltration grade.
Krepkin [43]	Case-Series, IV	9 (3/5)	Patients with shoulder pain with suspected rotator cuff pathology	SWE and MRI	SWE to examine tear status and correlated to MRI	There was a significant negative correlation between T2* and SWV in the lateral ROI (r = −0.86) and overall mean ROI (r = −0.90). There was significant positive correlation between T2 and measures of tear size in the lateral and mean ROIs (r range 0.71–0.77). There was significant negative correlation between SWV and tear size in the middle and mean ROIs (r range −0.79–−0.68).
Rosskopf [44]	Case-Control, III	44 (22/22)	44 patients with diseased supraspinatus muscle and 22 health volunteers	SWE and MRI	SWE to examine tear status and correlated to MRI	No significant difference in MTSWV was found based on tendon integrity. MTSWV varies significantly between patients with different degrees of retraction (*p* = 0.047).
Lawrence [45]	Cross-Sectional, III	22 (18/4)	22 patients with small- or medium-sized full-thickness supraspinatus tear	SWE and MRI	Correlating SWE estimated shear modulus to MRI findings	Estimated shear modulus was not significantly associated with tear size (*p* > 0.09), tear retraction (*p* > 0.20), occupation ratio (*p* > 0.11), or fatty infiltration (*p* > 0.30) under any testing condition.
Itoigawa [46]	Case-Series, IV	38	Patients with k-wn full-thickness supraspinatus tears	SWE and MRI	Correlating SWE estimated shear modulus to MRI findings	Ultrasound SWE can best predict the stiffness of the posterior deep muscles of supraspinatus musculotendinous unit (r = 0.69) Moderate correlation of SWE stiffness and Goutallier stage on MRI (r = 0.48).
Gilbert [47]	Cross-Sectional, III	42	Patients with history of rotator cuff tears	SWE and MRI	Correlating SWE estimated shear modulus to MRI/MRS findings	Correlation of SWV measured with SWE with the spectroscopic fat ratio was r = 0.82. SWV increased with higher fat/water ratio. Mean SWV was 1.81 m/s.
Contrast-Enhanced Ultrasound (CEU)						
Kunz [48]	Prospective Cohort, II	35	Patients following arthroscopic rotator cuff repair	Contrast- Enhanced US	CEU to examine muscle profusion of altered rotator cuff	Preoperative CEUS-based assessment of SSP perfusion significantly correlated with early postoperative shoulder function measured by Constant score, (r = 0.48) and tendon retear (r = 0.67).

**Table 2:** Imaging Protocols.

Study	Ultrasound Unit	Ultrasound Transducer	Ultrasound Positioning	MRI	MRI Sequences
Park [27]	HD11 Scanner (Phillips)	High frequency linear array probe (7.5–15 MHz)	Seated with sonographer behind patient	Not provided	Coronal turbo spin-echo T1, coronal T2 with fat suppression, oblique sagittal turbo spin-echo T1, oblique sagittal T2 with fat suppression, and axial proton density-weighted images with fat suppression.
Watanabe [28]	Pro-Sound a7 (Hitachi Aloka Medical)	Linear array probe (6.0–14.0 MHz)	Seated with sonographer behind patient	1.5 T (Intera Achieva Pulsar, Philips Medical Systems)	Sagittal oblique -n-fat-suppressed T2-weighted fast spin-echo (TR/TE, 2185–4968/100 ms)
Seo [29]	Acuson S2000 (Siemens)	Linear probe (9.0 MHz)	Seated with sonographer behind patient	Not provided	Oblique coronal proton density T1-weighted and T2-weighted fat saturated spin-echo images (TR/TE, 3300/14–95 ms), oblique coronal T1-weighted fat saturated spin-echo images (TR/TE, 777/12 ms), oblique sagittal T1-weighted spin-echo (TR/TE, 600/12 ms) T1-weighted spin-echo images (TR/TE, 600/12 ms)
Wall [30]	Elegra (Siemens), Antares (Siemens), iU22 (Philips), or E9 (GE)	Variable high-frequency linear array probe (7.5 to 15 MHz)	Seated with sonographer behind patient	Not provided	Axial spin-echo T1, axial fast spin-echo T2 with fat saturation, oblique coronal spin-echo T1, oblique coronal fast spin-echo T2 with fat saturation, oblique sagittal spin-echo T1, and oblique sagittal fast spin-echo T2 with fat saturation.
Khoury [31]	ATL 5000 HDI (Philips)	Linear probe (5–12 MHz)	Seated with sonographer behind patient	1.5 T (LightSpeed, GE or Avento, Siemens)	Sagittal oblique T1-weighted turbo spin-echo sequence (TR/TE, 525–535/10–15 ms) and coronal oblique T1-weighted turbo spin-echo sequence (TR/TE, 600–620/10–14 ms)
Chen, X [32]	Samsung Medison or Philips Electronics Tech-logy	LV3-14A Linear Probe (3–14 MHz) or XL14-3 Linear Probe (3–14 MHz)	Seated with sonographer behind patient	3.0 T (Siemens Skyra)	T1-weighted saggital and T1-weighted coronal (TE/TR 22/600 ms), Proton Density-weighted saggital (TE/TR 37/3800 ms), Proton Density-weighted coronal (TE/TR 42/3100 ms), Proton Density-weighted Transverse (TE/TR 73/3780 ms). The field of view set to 18 cm, and image sequences gained with a matrix acquisition range of 320 × 256. Slice thickness was 4 mm.
Guerini [33]	Aplio (Toshiba)	High frequency linear probes (7–15 MHz and 6–12 MHz)	Seated with sonographer behind patient	1.5 T (Siemens or GE)	T1 weighted sagittal image and T2 weighted spatial planes with fat saturation. T1 weighted with fat saturation and intrave-us gadolinium enhancement
Chen, D [34]	LOGIC 500 unit (GE)	Linear probe (6–13 MHz)	Not reported	1.5 T Symphony Tim and Magnetom Area (Siemens)	Fast spin-echo proton-weighted sequence (TE/TR 25/3000 ms) in an axial and oblique sagittal plane with oblique coronal slices perpendicular and parallel to the course of the tendon of the supraspinatus, respec- tively, and FSE T2-weighted fat- suppressed images (TE/TR 100/4500 ms) in axial, oblique sagittal, and oblique coronal planes
Rutten [35]	Aplio (Toshiba)	Linear array probe (7.5–14 MHz)	Seated with sonographer behind patient	1.5 T (Signa Horizon, GE)	MRI: Oblique coronal T2-weighted with fat suppression and T1-weighted turbo spin-echo, oblique sagittal T2-weighted turbo spin-echo without fat suppression and transverse T1-weighted turbo spin-echo. MRA: 3D-gradient T1- weighted oblique coronal and axial. 3D imaging (TR/TE, 51/7 ms) and coronal T2-weighted turbo spin-echo, sagittal T2-weighted turbo spin-echo and coronal T1-weighted turbo spin echo images with fat suppression
Ueda [36]	F450AX Bettius; (Fukuda Denshi Co.)	38 mm linear array probe (7 MHz)	Seated with sonographer behind patient	Not provided	TI weighted (TR/TE, 3500/98 ms) and T2 weighted oblique sagittal
Kretic [37]	Aloka Prosound Alpha 6 (Hitachi Healthcare)	Linear probe (5–13 MHz)	Seated with sonographer behind patient	1.5 T (Siemens Avanto)	T1 weighted (TR/TE, 472/15 ms)
Yi [38]	LOGIQ E9 (GE Healthcare)	Linear probe (5–12 MHz)	Seated with sonographer behind patient	1.5 T (Siemens)	T1- wieghted
Chen, P [39]	Acuson S2000 (Siemens Healthcare)	Linear array probe (4–9 MHz)	Seated with sonographer behind patient	1.5 T (Signa Horizon LX, GE Healthcare)	Axial proton-density-weighted fast spin-echo with a fat suppression (TR/TE, 2700–4800/25–40 ms), coronal oblique proton-density-weighted fast spin-echo with and without fat suppression (TR/TE, 2700–4800/25–40 ms), and oblique proton-density-weighted fast spin-echo with and without fat suppression (TR/TE, 2700–4800/25–40 ms).
Huang [40]	Aplio i800 ultrasound system (Ca-n Medical)	Linear probe (5–18 MHz)	Seated with sonographer behind patient	1.5 T (Magnetom Essenza; Siemens Healthcare)	Proton density (PD)-weighted turbo spin-echo coronal oblique, axial, and sagittal oblique planes and T1-weighted turbo spin echo coronal oblique and sagittal oblique planes
Ruder [41]	Acuson S3000 (Siemens)	Linear probe (8 MHz)	Seated with sonographer behind patient	1.5 T	Axial and sagittal-oblique fat suppressed proton density, coronal-oblique and sagittal-oblique T1-weighted, and coronal-oblique fat-suppressed T2-weighted
Jeong [42]	Aplio i800 (Ca-n Medial System)	Linear probe (18 MHz)	Seated with sonographer behind patient	3.0 T (Skyra, Siemens Healthineers	Axial, coronal, and sagittal proton-density fat-saturation, T2-weighted axial, sagittal, and coronal, and T1 weighted sagittal (TR/TE, 632–689/12 ms)
Krepkin [43]	Acuson S3000 scanner (Siemens Medical Solutions)	Linear prove (9 MHz)	Seated with sonographer behind patient	3.0 T (Magnetom Skyra, Siemens Healthcare)	Coronal fat-suppressed T2-weighted turbo-spin echo, sagittal T1-weighted turbo-spin echo, sagittal fat-suppressed T2-weighted turbo-spin echo or axial fat suppressed T2-weighted turbo-spin echo. T2 mapping sequences consisted of 2D multi-echo spin echo sequence with five echoes and T2* mapping sequences consisted of 2D multi-echo spoiled gradient echo (GRE) sequence with six echoes
Rosskopf [44]	Acuson S3000 scanner (Siemens Medical Solutions)	Linear array probe (4–9 MHz)	Seated with sonographer behind patient	1.5 T and 3.0 T	Coronal oblique T1-weighted fast spin-echo sequence with fat suppression (TR/TE, 500/12 ms), sagittal oblique T1-weighted spin-echo sequence (TR/TE, 550/12), transverse three-dimensional water-excitation true fast imaging spin-echo sequence with steady-state precession (TR/TE, 9.44/3.88), coronal oblique proton density–weighted spin-echo with fat suppression (TR/TE, 3000/53 ms), and sagittal oblique T2-weighted spin-echo with fat suppression (TR/TE, 4340/69 ms).
Lawrence [45]	Siemens ACUSON S3000	9L4 linear probe	Seated with sonographer behind patient	1.5 T	Axial and oblique sagittal proton density images with fat suppression, oblique coronal and oblique sagittal T1-weighted without fat suppression, and oblique coronal T2-weighted with fat suppression.
Itoigawa [46]	Aixplorer System	SL10-2 linear array probe	Seated with sonographer behind patient	Not provided	Not provided
Gilbert [47]	Siemens ACUSON S3000	Linear array probe (10 MHz) the SWV was assessed with virtual touch tissue imaging quantification (VTIQ)	Seated with sonographer behind patient	3.0 T (Skyra, Siemens, German)	T1-weighted images (TR/TE, 653 ms/12 ms), FOV 180 mm and for the SPLASH Sequence, (TR/TE = 35/5–25 ms), FOV 278 mm. Slices were 5 mm for the SPLASH technique and 3 mm for the standard MRI
Kunz [48]	Siemens ACUSON S3000	9L4 linear probe	Seated with sonographer behind patient	Not provided	T1-weighted Y-planeview of the scapula, proton-density weighted coronal, proton-density weighted sagittal Y-planeview, coronary T2, and proton-density weighted

**Table 3:** GRADE ratings for the quality of articles based on the risk of bias, imprecision, inconsistency, indirectness, and publication bias.

Study	(1) Inconsistency	(2) Indirectness	(3) Imprecision	(4) Publication Bias	(5) Risk of Bias	Overall Quality Score (0–5)
Park [27]	No	No	Yes	No	Yes	3
Watanabe [28]	No	No	No	No	Yes	4
Seo [29]	No	No	Yes	No	Yes	3
Wall [30]	No	No	Yes	No	No	4
Khoury [31]	No	No	No	No	Yes	4
Chen, X [32]	No	No	Yes	No	No	4
Guerini [33]	No	No	Yes	No	Yes	3
Chen, D [34]	No	No	Yes	No	Yes	3
Rutten [35]	No	No	No	No	Yes	4
Ueda [36]	No	No	No	No	No	4
Kretic [37]	No	No	Yes	No	No	4
Yi [38]	No	Yes	No	No	Yes	3
Chen, P [39]	No	No	No	No	No	5
Huang [40]	No	No	No	No	No	5
Ruder [41]	No	No	Yes	No	Yes	3
Jeong [42]	No	No	No	No	Yes	4
Krepkin [43]	No	No	No	No	Yes	4
Rosskopf [44]	No	No	Yes	No	Yes	3
Lawrence [45]	No	No	No	No	Yes	4
Itoigawa [46]	No	No	No	No	No	5
Gilbert [47]	No	No	No	No	Yes	4
Kunz [48]	No	Yes	Yes	No	Yes	2

**Table 4:** Summary of reporting recommendations.

Participant Demographics Number of participants; Basic demographics (gender/sex, height, weight, age, ethnicity, and BMI); Relevant Past Medical History (diag-sis, surgical interventions, diabetes, cancer, etc.); Recent medical treatment (injections, physical therapy, duration/frequency of treatment, etc.).
Study Design and Procedure Study design (Randomized Controlled Trial, Cross-sectional, Prospective, Retrospective, etc.); Detailed data collection procedures; Number of study visits; Number and list of structures examined; Number of images captured per structure (for each imaging mode; B-mode, SWE, etc.); Average depth, range, and other relevant adjustments.
Pre-Collection Information Describe any restrictions placed on research subjects (Recent exercise, fasted for blood draws, etc.); Warm up or stretching routine prior to data collection; Verbal or written instructions given to participants prior to data collection.
Ultrasound Imaging Protocol Imaging system settings (musculoskeletal, breast, nerve, etc.); Device brand(s), model, and manufacturer headquarters; Probe(s) parameters; Upper and lower limits of system; Upper and lower limits of ultrasound probe; Detailed Subject and Tester positioning: o Position of body, limb, joint, etc. with illustrations; o Cueing provided to patient throughout data collection (relaxed or contracted muscles); o External support for positioning (tables, chairs, belts, straps, pads, etc.). Structures examined; Limb examined, including arm dominance; Anatomical references and landmarks utilized to standardize protocol; Illustrations for probe placement during data collection; How probe was secured (manual or external stabilizer); Coupling agent utilized, including amount or pad thickness; Degree of pressure applied to probe.
Operator Training Number of operators; In detail, describe formal training and experience of each operator.
Regions of Interest Describe each region of interest and how each were standardized; Number, size, and depth (superficial, intermediate, deep, etc.) of each region of interest.
Imaging Results
If multiple measurements, report reliability results (inter- and intra-class correlation coefficients); Provide details of results for each ROI within each structure (cross-sectional area measured in cm^3^, shear modulus in kPa, shear velocity in m/s, etc.); Mean and standard deviations; Post-image Processing software (Matlab, Dicom, etc.).

## Data Availability

All data underlying this article will be shared upon reasonable request to the corresponding authors.
